# Laparoscopic cytoreductive surgery and hyperthermic intraperitoneal chemotherapy: a prospective clinical trial and comparative analysis

**DOI:** 10.1007/s00464-022-09589-w

**Published:** 2022-12-07

**Authors:** Chae Yun Cho, Jula Veerapong, Joel M. Baumgartner, James D. Murphy, Andrew M. Lowy, Kaitlyn J. Kelly

**Affiliations:** 1 Department of Surgery, University of California, San Diego, 3855 Health Sciences Drive, Mail Code 0987, San Diego, La Jolla, CA 92093 USA; 2 Department of Radiation Medicine, University of California, San Diego, La Jolla, USA

**Keywords:** Hyperthermic intraperitoneal chemotherapy, Cytoreductive surgery, Minimally-invasive surgery, Peritoneal surface malignancy

## Abstract

**Background:**

Open cytoreductive surgery (CRS) and hyperthermic intraperitoneal chemotherapy (HIPEC) is associated with high morbidity, which limits the degree to which patients may benefit from this therapy. This study aimed to determine the feasibility of laparoscopic CRS/HIPEC.

**Methods:**

This was a single institution prospective clinical trial and comparative study using historical controls. Patients with histologically confirmed peritoneal surface malignancy (PSM) of appendiceal, colorectal, ovarian, or primary peritoneal origin, peritoneal carcinomatosis index (PCI) $\le$ 10 were eligible.

**Results:**

Clinical trial: 18 patients (median age 57 years, 39% female) with appendiceal (15) or colorectal (3) primary PSM underwent laparoscopic CRS/HIPEC. Median and range outcomes were: operative time 219 min (134–378), EBL 10 mL (0–100), time to return to bowel function 3 days (1–7), duration IV narcotic use 3 days (1–8), length of stay 6 days (3–11). All patients had a complete cytoreduction (CC-score 0). Three (17%) experienced minor morbidity, with no major morbidity or mortality. Median DFS and OS were not reached with median follow-up of 48 months. Comparative analysis: Laparoscopic approach associated with reduced time to return of bowel function (3 versus 4 days, *p* = 0.001), length of stay (8 versus 5 days, *p* < 0.001), and morbidity (16% versus 42%, *p* = 0.008). Independent predictors of DFS included prior chemotherapy (HR 5.07, 95% CI 1.85, 13.89; *p* = 0.002), and CC-score > 0 (HR 3.31, 95% CI 1.19, 9.41; *p* = 0.025), but not surgical approach. CC-score > 0 was the only independent predictor of OS (HR 10.12, 95% CI 2.16, 47.30, *p* = 0.003).

**Conclusions and relevance:**

Laparoscopic CRS/HIPEC should be considered for patients with PSM with low-volume disease, including those with adenocarcinoma histology.

**Trial registration:**

Clinicaltrials.gov; NCT02463877.

Cytoreductive surgery (CRS) and hyperthermic intraperitoneal chemotherapy (HIPEC) is the standard-of-care treatment modality for selected patients with peritoneal surface malignancy (PSM). It has been studied extensively in appendiceal and ovarian primary cancers, as well as colorectal, primary peritoneal, and gastric cancer. A drawback of this therapy is the invasive nature of the procedures and associated morbidity. Open CRS/HIPEC is typically performed through a xiphoid-to-pubis incision and carries a significant risk of postoperative morbidity, on the order of 33 to 63% [1]. Laparoscopic CRS/HIPEC has been investigated as a means of decreasing the procedure-related morbidity. The excellent exposure and visibility afforded by laparoscopy and the broad range of oncologic procedures that can be safely performed minimally invasively, make this approach feasible for patients with limited peritoneal disease.

Several studies have demonstrated decreased postoperative morbidity and improvements in short-term postoperative outcomes, such as blood loss, and length of stay, with a laparoscopic approach [2–5]. These studies have concluded that laparoscopic CRS/HIPEC is safe and feasible for patients with a peritoneal carcinomatosis index (PCI) of < 10. Limitations of these studies include relatively short follow-up with little data on long-term oncologic outcomes, small numbers of patients, and inclusion of only patients with low-grade disease.

Patients with low-grade PSM are those who are likely to have the best long-term outcomes following CRS/HIPEC. Additionally, these patients are least likely to have their oncologic outcomes affected by postoperative complications, as these are not invasive cancers [1]. Patients with more aggressive, higher-grade malignancies, for example colorectal and gastric cancers, are those for whom CRS/HIPEC is more controversial as the risk / benefit ratio is less favorable [6]. These patients have a higher risk of extraperitoneal disease progression and early peritoneal recurrence, and the benefit of aggressive CRS/HIPEC is not as certain. In addition, these patients are more likely to have poorer oncologic outcomes if significant postoperative complications occur [1]. It is these patients who would benefit most from the decreased morbidity of laparoscopic CRS/HIPEC.

We conducted a prospective clinical trial of laparoscopic CRS/HIPEC for patients with PSM of appendiceal or colorectal primary origin, inclusive of adenocarcinoma histology, with PCI $\le$ 10. The aim of the study was to determine postoperative morbidity and mortality, as well as disease free and overall survival, following laparoscopic CRS/HIPEC. In this manuscript, we report the findings of this trial with mature follow-up data. In addition, we report a comparative analysis of laparoscopic versus open CRS/HIPEC on patients with PSM of gastrointestinal primary origin with PCI $\le$ 10.

## Methods

### Clinical trial

#### Design

This was an IRB-approved, prospective phase II clinical trial of laparoscopic CRS/HIPEC (NCT02463877). Data may be shared in a de-identified form upon request, with an IRB-approved research plan. The primary endpoint of the study was 30-day postoperative morbidity, defined according to the validated system proposed by Clavien [7]. Briefly, a complication was any deviation from the normal postoperative course. Grade I were deviations that did not require intervention for treatment. Grade II required pharmacologic intervention. Grade III required surgical, endoscopic, or radiologic intervention. Grade IV were life-threatening events requiring intensive care for management. Grade V were deaths. Minor morbidity was defined as grade I or II complications, and major morbidity was grade III or higher. Secondary endpoints included completeness of cytoreduction score (CC-score), time to return of bowel function, duration of intravenous narcotic use, length of hospital stay, disease free survival, and overall survival (both determined from the date of surgery).

#### Patients

Eligibility criteria included: age $\ge$ 18 years of age, histologically confirmed peritoneal surface malignancy of appendiceal, colorectal, ovarian, or primary peritoneal origin, estimated peritoneal carcinomatosis index (PCI) of $\le$ 10, Eastern Cooperative Oncology Group (ECOG) performance status of 0–2, absence of extraperitoneal metastasis (including hepatic parenchyma), and medical fitness for surgery. Exclusion criteria included extensive intra-abdominal adhesions preclusive of complete laparoscopic exploration of the peritoneum, disease not amenable to laparoscopic removal, as well as active pregnancy or lactation, concurrent second malignancy requiring systemic therapy, and concurrent psychiatric disorders or other conditions that would preclude a patient from meeting study requirements.

Screening was performed at the time of presentation to surgical oncology clinic. Of 34 patients screened and consented for the study, 16 were deemed ineligible due to PCI > 10 (10) or disease not amenable to laparoscopic excision (6). Eighteen patients were eligible and proceeded on the study.

#### Surgery

Laparoscopic CRS/HIPEC was performed via a closed technique. Patients were positioned split-leg with adequate stabilization to allow for positional changes including steep Trendelenburg and reverse Trendelenburg positioning, well as side-to-side tilting. Thorough, systematic exploration for disease was performed starting with the upper abdomen, using a 30 degree laparoscope. All parietal peritoneal surfaces were inspected including in the pelvis. The gastrocolic ligament was incised and the lesser sac was inspected. The ligament of Treitz was identified and inspected and the small bowel was run from there to the ileocecal valve, examining the serosa and the mesenteric surfaces. Finally, the intraperitoneal colon was inspected as were the para-colic gutters.

**Fig. 1:**
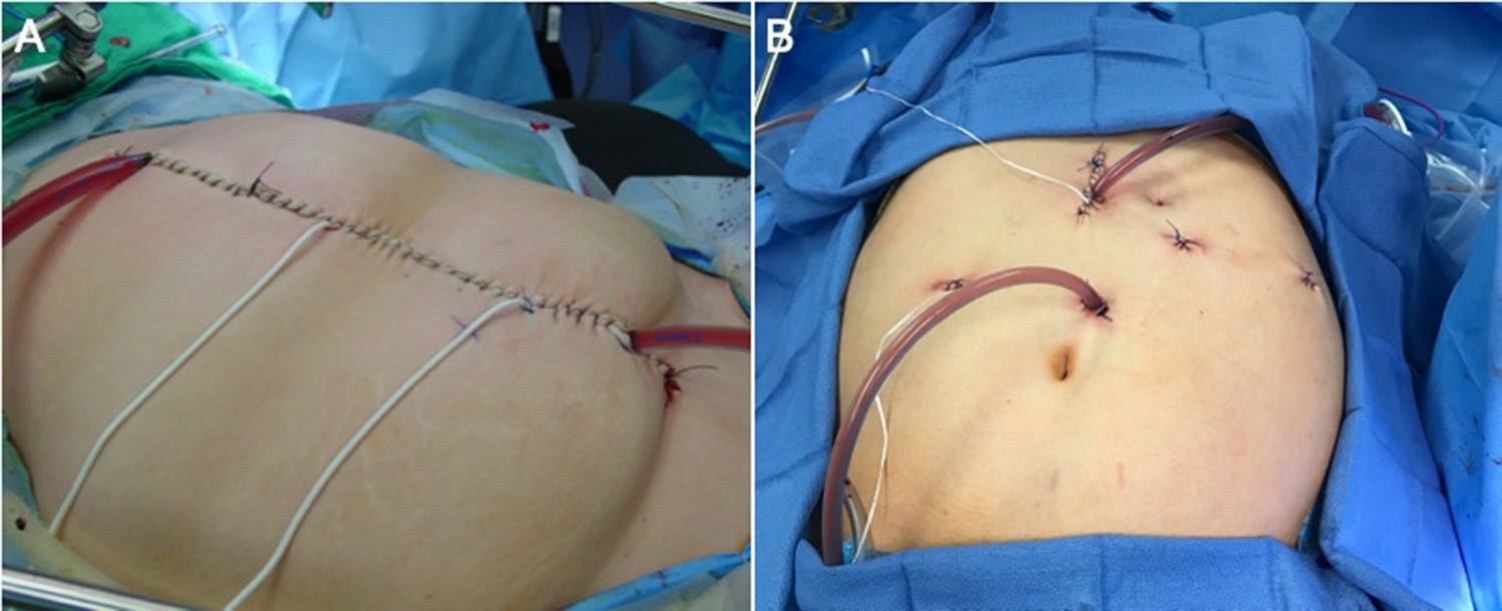
Operative photos showing standard open incision for CRS/HIPEC extending from xiphoid process to pubic symphysis (**A**) compared to incisions for a laparoscopic CRS/HIPEC procedure (**B**)

Once all sites of disease were identified, laparoscopic cytoreduction was performed, including selective peritonectomy. Specimens were placed in a sterile bag and removed via an existing port site, or via an enlarged port site lined with a wound protector. HIPEC catheters were then inserted via the existing port sites and/or the extraction site. HIPEC was performed with mitomycin C (MMC) as previously described [8]. When HIPEC was completed, the peritoneal cavity was perfused with 3 L of sterile saline and the catheters were carefully removed. Any gastrointestinal reconstruction was then performed. Typical laparoscopic and open incisions are shown in Fig. 1A and B, respectively.

#### Statistics

A balanced two-stage clinical trial design was utilized with a primary endpoint of 30-day morbidity following minimally invasive CRS/HIPEC. The rate of 30-day morbidity following open HIPEC was approximately 43% (null hypothesis). A clinically meaningful reduction in postoperative morbidity was determined to be 23%, bringing 30-day morbidity down to 15% (alternate hypothesis). A sample size of 18 patients accrued in two stages to yield a power of 81% to detect a 28% decrease in the rate of toxicity (from 43 to 15%) assuming *α* = 0.05. In stage I, 9 patients were enrolled. If 2 or less patients were to experience postoperative complications within 30 days of HIPEC the study would proceed to stage 2. If 3 or more patients experience postoperative complications, the study would be terminated early due to futility. In stage II, an additional 9 patients would be enrolled. If 4 or fewer total patients were to experience 30-day morbidity, the null hypothesis would be rejected.

Variables collected included: Age, body mass index (BMI), gender, primary tumor site, histologic diagnosis, peritoneal carcinomatosis index (PCI), operative time, estimated blood loss (EBL), number of organs resected, number of anastomoses, completeness of cytoreduction (CC) score, time to first flatus, length of intensive care unit (ICU) stay, length of hospital stay, 30-day morbidity, 30-day mortality, disease free survival (DFS), and overall survival (OS). Descriptive statistics were utilized to assess categorical and continuous variables. Mean and median DFS and OS were determined with the Kaplan–Meier test.

### Comparative study

Once the phase II trial was completed, laparoscopic CRS/HIPEC was offered to all patients with PSM with PCI $\le$ 10, regardless of primary site or histology. The same surgical approach as described for the clinical trial was continued. For patients with gastric or small bowel primary tumors, HIPEC was performed with MMC (10 mg/L perfusate) and Cisplatin (50 mg/m^2^). For peritoneal mesothelioma, HIPEC was performed with Cisplatin (50 mg/m^2^) and doxorubicin (15 mg/m^2^). For ovarian cancer, HIPEC was performed with single-agent Cisplatin (100 mg/m^2^). A prospectively maintained database of patients who underwent CRS/HIPEC (including the clinical trial patients) was queried for patients with PCI $\le$ 10. Those who underwent laparoscopic versus open CRS/HIPEC were compared. Laparoscopic-converted-to-open cases were treated as open. Additional variables that were collected for the comparative analysis included inpatient and 60-day morbidity.

#### Statistics

Patients with a PCI $\le$ 10 who underwent laparoscopic versus open CRS/HIPEC were compared. The Chi-square test was used to compare categorical variables between the two groups and the Wilcoxon Rank Sum test was used for continuous variables. Statistical significance was set at *p* value < 0.050. The Kaplan–Meier test and Cox Proportional Hazards test were used to identify variables associated with DFS and OS. Multivariate models were constructed based on results of univariate analyses, with variables associated with DFS and OS with a *p* value < 0.200 on univariate analysis included. All combinations of variables in multivariate models were evaluated for interactions.

## Results

### Trial patients

A total of 18 patients were enrolled and treated with laparoscopic CRS/HIPEC. The median age was 57 years and 39% of patients were female. The primary tumor site was the appendix in 15 cases and the colon in 3. Of the appendiceal primary patients, 8 had LAMN, 2 had mucinous adenocarcinoma, and 5 had poorly differentiated adenocarcinoma with signet ring cells. The 3 patients with colon primary tumors had moderately differentiated adenocarcinoma. Four of the 10 patients (40%) with adenocarcinoma histology had systemic chemotherapy prior to surgery, and 3 (30%) received adjuvant systemic therapy. Three patients refused systemic treatment. At the time of CRS/HIPEC, 9 patients had residual adenocarcinoma, 2 had persistent LAMN, 1 had persistent low-grade carcinoma peritonei, and 6 had only fibrous tissue or acellular mucin remaining. The median PCI was 3 (range 0–10). The median operative time was 219 min (range 134–378) and median EBL was 10 mL (range 0–100). The most common procedures performed were omentectomy (*n* = 13), selective peritonectomy including parietal peritonectomy and removal of visceral peritoneal implants (*n* = 16), and right hemicolectomy (*n* = 5). Additional procedures performed were oophorectomy (*n* = 1), appendectomy (*n* = 2), and ablation of small bowel mesenteric and liver surface lesions (*n* = 2). All patients had a complete cytoreduction (CC-score 0). Patient baseline, disease-related, operative, and postoperative characteristics are summarized in Table 1.

**Table 1:** Baseline, operative, and postoperative variables of 18 patients who underwent laparoscopic CRS/HIPEC on a prospective clinical trial

	Total patients (*n* = 18)
Age	57 (29–78)
BMI	24.7 (17.5–38.1)
Female sex	7 (39)
Primary site	
Appendix	15 (83.3)
Colon	3 (16.6)
Histology at CRS/HIPEC	
Adenocarcinoma	9
Low-grade appendiceal mucinous neoplasms (LAMN)	2
Low-grade carcinoma peritonei	1
Fibrous tissue/acellular mucin	6
Peritoneal Carcinomatosis Index	3 (0–10)
Operative time (min)	219 (134–378)
Number organs resected	1.5 (0–3)
Number of anastomoses	0 (0–2)
Estimated blood loss (mL)	10 (5–100)
Time to flatus	3 (1–7)
Days of IV narcotic	3 (1–8)
Length of stay	6 (3–11)
Length of ICU stay	0 (0–2)
30-Day morbidity	3
30-Day morbidity grade	
1–2	3
3–4	0
60-Day morbidity	
1–2	1
3–4	0

**Fig. 2:**
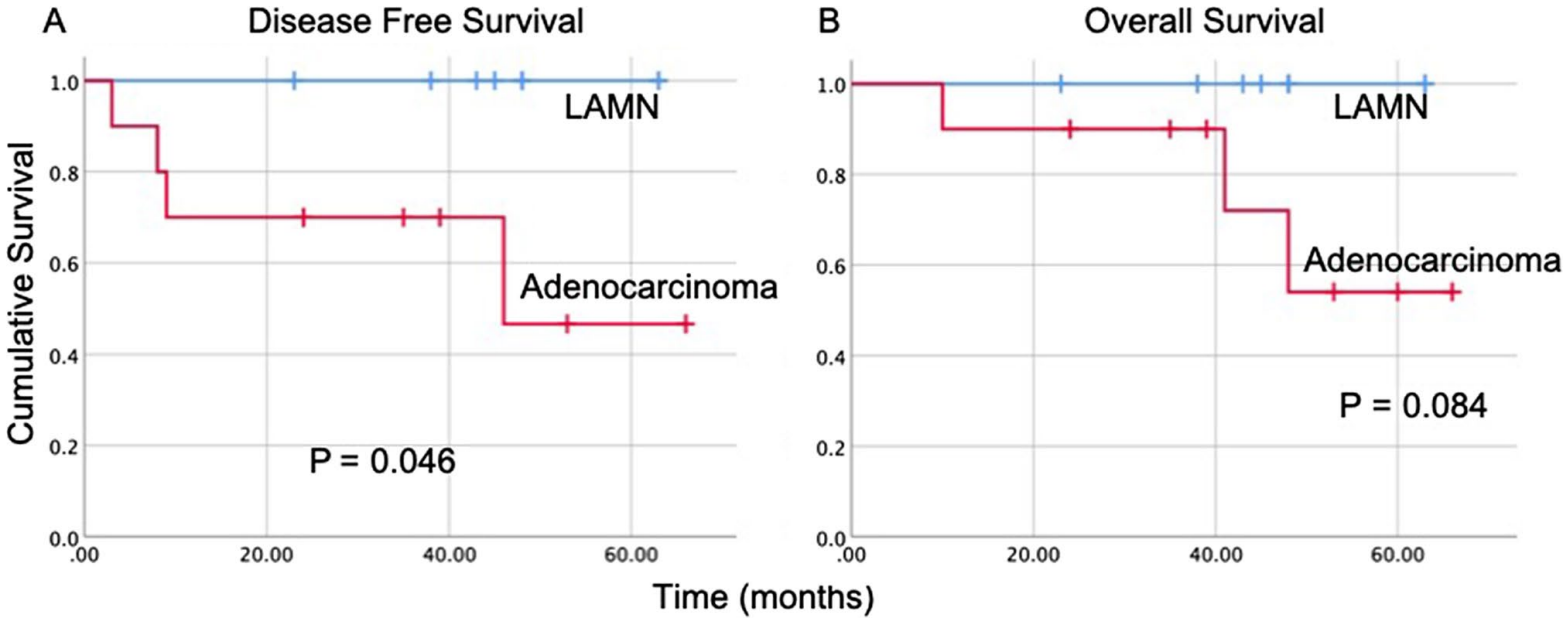
Disease free (**A**) and overall survival (**B**) curves for 18 patients who underwent laparoscopic CRS/HIPEC on a prospective clinical trial. At a median follow-up of 48.0 months, there were no recurrence or death events in the subset of patients with LAMN. Median disease free and overall survival were not reached, but median DFS was significantly worse in patients with adenocarcinoma histology compared to those with LAMN

The median time to return to bowel function was 3 days (range 1–7), intravenous narcotic use was 3 (range 1–8), and length of stay was 6 (range 3–11). Three patients (17%) experienced 30-day morbidity, all of which were minor (one patient had urinary tract infection, one had leukopenia requiring GM-CSF, and one had postoperative laryngospasm requiring temporary BIPAP). There was no major morbidity or postoperative mortality. The median follow-up for trial patients was 48.0 months (95% CI 33.4, 57.3). During the follow up time, 4 patients experienced recurrence at 5.2, 8.0, 9.4 and 46.0 months, all of whom had adenocarcinoma histology. Three of these 4 patients died of disease. There were no recurrence events or deaths in the patients with LAMN (Fig. 2). Median DFS and OS were not reached.

### All patients

From January of 2014 to August 2020, a total of 52 patients underwent laparoscopic exploration for possible laparoscopic CRS/HIPEC, including the 18 trial patients. The procedure was converted to open in 21 cases (40%), because of underestimation of the PCI (*n* = 18) or disease distribution not amenable to laparoscopic removal (*n* = 3). Laparoscopic CRS/HIPEC was performed in 31 patients. These patients were compared to 160 with PCI $\le$ 10 who underwent open CRS/HIPEC during the same time period.

A comparison of baseline, disease-related, operative, and postoperative variables between the laparoscopic and open groups is shown in Table 2. There were no statistically significant differences in age, gender, or BMI between the laparoscopic and open groups. There was a statistically significant difference in the distribution of primary tumor sites between the groups, with patients in the open group more likely to have peritoneal or ovarian primary tumors (13% versus 0, *p* = 0.023), but there was no difference in rate of adenocarcinoma histology (58% laparoscopic versus 61% open, *p* = 0.800). Patients in the laparoscopic group had a lower median PCI (3 versus 7, *p* < 0.001). There was no difference in the rate of complete cytoreduction between the two groups, but operative time and EBL were greater in the open group (median operative time 366 min versus 277 min, *p* = 0.013; median EBL 100 mL versus 25 mL, *p* < 0.001). Time to return of bowel function after surgery was shorter in the laparoscopic group (median 3 days versus 4 days, *p* = 0.001), as was hospital length of stay (5 days versus 8 days, *p* < 0.001). 30-day postoperative morbidity was significantly less in the laparoscopic group (16% versus 42%, *p* = 0.008). This difference was accounted for by lower minor morbidity, which was 13% in the laparoscopic group versus 34% in the open group (*p* = 0.012). 30-day major morbidity was low in both groups (4% for laparoscopic and 8% in the open group) and was not statistically significantly different (*p* = 0.498). There were no differences in 60-day minor or major morbidity.

**Table 2:** Comparison of baseline demographic, disease-related, operative, and postoperative variables for patients with PCI $\le$ 10 treated by laparoscopic or open CRS/HIPEC

Variable	Laparoscopic (*N* = 31)	Open (*N* = 160)	*p*
Female gender	16 (52%)	108 (68%)	0.090
Age at surgery (years)	57 (29–78)	55 (26–86)	0.624
Body Mass Index	25.7 (17.8–38.6)	25.0 (16.7–44.1)	0.674
Primary site			0.008
Appendix	24 (77%)	68 (43%)	
Colon/rectum	5 (16%)	58 (36%)	
Peritoneum (mesothelioma)	0	13 (8%)	
Ovary	0	11 (7%)	
Other GI (gastric and small bowel)	2 (8%)	10 (6%)	
Primary histology			0.080
Low grade mucinous neoplasm	13 (42%)	41 (26%)	
Adenocarcinoma	18 (58%)	98 (61%)	
Mesothelioma	0	12 (7%)	
Ovarian carcinoma	0	9 (6%)	
Prior chemotherapy	9 (29%)	106 (66%)	< 0.001
Postoperative chemotherapy	6 (19%)	30 (19%)	0.924
Peritoneal Carcinomatosis Index	3 (0–10)	7 (0–10)	< 0.001
Complete Cytoreduction Score			0.788
0	30 (97%)	148 (93%)	
1	1 (3%)	10 (6%)	
2	0	2 (1%)	
Operative time (min)	277 (146–112)	366 (194–866)	0.001
Estimated blood loss (mL)	25 (5–250)	100 (10–1300)	< 0.001
Time to return of bowel function (days)	3 (1–5)	4 (1–16)	0.001
Length of stay (days)	5 (3–11)	8 (3–68)	< 0.001
Any 30-day morbidity	5 (16%)	67 (42%)	0.008
Minor 30-day morbidity	4 (13%)	55 (34%)	0.012
Major 30-day morbidity	1 (4%)	12 (8%)	0.498
Any 60-day morbidity	3 (12%)	39 (24%)	0.147
Minor 60-day morbidity	4 (13%)	34 (21%)	0.491
Major 60-day morbidity	0	5 (3%)	0.319
60-Day readmission	1 (3%)	22 (14%)	0.151

The median follow-up for the entire group was 24.0 months (38.4 months for the laparoscopic group versus 17.0 months for the open group). Table 3 depicts predictors of DFS and OS in the cohort of patients with gastrointestinal primary tumors (appendix, colorectal, and stomach / small bowel). On univariate analysis, open surgical approach, non-appendiceal primary tumor site, adenocarcinoma histology, prior chemotherapy, EBL, and operative time were significantly associated with DFS. On multivariate analysis adjusting for these variables, surgical approach was not an independent predictor of DFS. Independent predictors of DFS included prior chemotherapy (HR 5.07, 95% CI 1.85, 13.89; *p* = 0.002), and CC-score > 0 (HR 3.31, 95% CI 1.19, 9.41; *p* = 0.025). Median OS was not reached. On univariate analysis, gastric / small bowel versus appendiceal primary site, prior chemotherapy, and CC-score > 0 were statistically significantly associated with poorer OS. On multivariate analysis, CC-score > 0 was the only independent predictor of poorer OS (HR 10.12, 95% CI 2.16, 47.30, *p* = 0.003) (Table 3).

**Table 3:** Factors associated with disease free and overall survival in 170 patients with PSM of GI primary origin with PCI $\le$ 10 treated with CRS/HIPEC

Univariate analysis				Multivariate analysis		
Variable	HR	95% CI	*p*	HR	95% CI	*p*
Disease free survival						
Open approach	2.69	1.17, 6.18	0.020	–	–	–
Primary site	–	–	< 0.001	–	–	–
Appendix	REF	–	–	–	–	–
Colon and rectum	4.56	2.32, 8.98	< 0.001	–	–	–
Gastric and small bowel	7.75	2.42, 24.74	0.001	–	–	–
Adenocarcinoma histology	11.211	3.45, 36.39	< 0.001	–	–	–
Prior chemotherapy	10.12	4.20, 24.80	< 0.001	5.07	1.85, 13.89	0.002
PCI	1.003	0.932, 1.141	0.552	–	–	–
EBL	1.001	1.000, 1.002	0.022	–	–	–
Operative time (min)	1.003	1.001, 1.005	0.002	–	–	–
CC-Score > 0	2.50	0.89, 6.99	0.084	3.31	1.17, 9.41	0.025
Overall survival						
Open approach	1.75	0.45, 6.85	0.407	–	–	–
Primary site	–	–	0.142	–	–	–
Appendix	REF	–	–	–	–	–
Colon and rectum	1.29	0.36, 4.63	0.697	–	–	–
Gastric and small bowel	9.56	1.01, 90.15	0.049	–	–	–
Adenocarcinoma Histology	6.94	0.89, 54.39	0.065	7.87	0.97, 63.73	0.053
Prior Chemotherapy	3.81	0.99, 14.63	0.052	–	–	–
PCI	1.15	0.92, 1.44	0.214	–	–	–
EBL	1.001	0.999, 1.003	0.506	–	–	–
Operative Time (min)	1.004	1.001, 1.008	0.025	–	–	–
CC-Score > 0	7.42	1.91, 28.87	0.004	8.87	2.10, 37.43	0.003

The comparative analyses were repeated on an intention-to-treat basis comparing the 52 patients who underwent laparoscopic or laparoscopic-converted-to-open CRS/HIPEC to 139 who had planned open CRS/HIPEC. The findings were similar with significantly reduced PCI, EBL, time to return of bowel function, LOS, and overall 30-day morbidity in the laparoscopic group (data not shown). The only independent predictor of DFS on this analysis was receipt of prior chemotherapy (HR 6.47, 95% CI 2.18, 19.23, *p* = 0.001). Similar to the previous analysis, when adjusting for histology, primary site, prior chemotherapy, operative time, and PCI, the only independent predictor of poorer OS in this intention-to-treat analysis was CC-score > 0 (HR 8.87, 95% CI 2.10, 37.43, *p* = 0.003).

## Discussion

The primary advantage of minimally invasive CRS/HIPEC is decreased morbidity as shown in this and other studies. An additional theoretical advantage that is not easily measured is decreased adhesion formation. These benefits are arguably most important to patients with aggressive malignancies who are most likely to need additional therapies, and in whom significant operative complications have the greatest impact. Despite this, minimally invasive CRS/HIPEC has only been prospectively studied in patients with low-grade disease [3–5]. Laparoscopic HIPEC without CRS has been investigated as a treatment for patients with PSM from aggressive gastrointestinal primary sites, such as gastric and colorectal cancers. [9] Only one study to date evaluated laparoscopic CRS/HIPEC for malignant PSM [10]. This was a retrospective analysis comparing laparoscopic versus open HIPEC for patients with PCI < 10 with no large tumors, diaphragm involvement, or multifocal mesenteric lesions. The authors reported improved short-term outcomes with the laparoscopic approach, but did not have long-term follow-up.

The clinical trial portion of this study demonstrated that patients with PSM are eager for minimally invasive CRS/HIPEC. This resulted in rapid accrual, despite a high rate of screen failure (53%). The rate of minor morbidity was 17% and there was no major morbidity. The long-term follow up for the trial patients was excellent, and median DFS and OS were not reached. The trial findings impacted clinical decision-making at our institution and we continued to offer laparoscopic CRS/HIPEC to eligible patients following its completion.

**Fig. 3:**
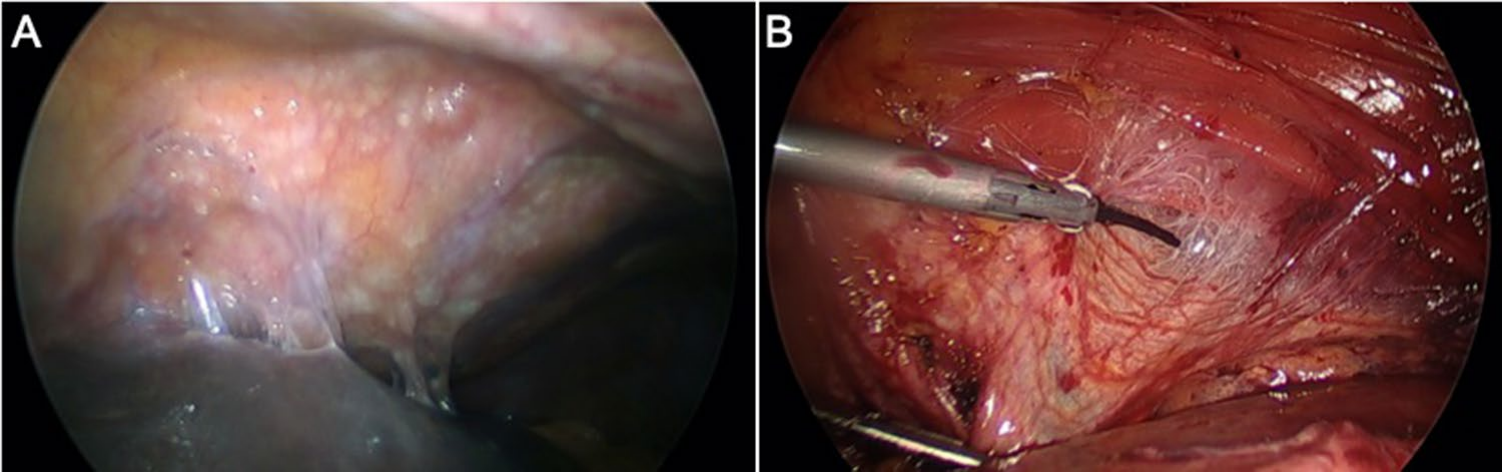
Image of peritoneal disease on the left diaphragm peritoneum (**A**). Diaphragmatic peritonectomy being performed laparoscopically with a harmonic scalpel with excellent hemostasis (**B**)

The findings of the clinical trial were similar in the comparative analysis, with decreased 30-day morbidity, length of stay, and EBL. EBL was possibly less due to the lower burden of disease in the laparoscopic group, but also potentially due to the technique of peritonectomy. Laparoscopic peritonectomy using a fine energy-sealing device can be performed with minimal blood loss as depicted in (Fig. 3). Open peritonectomy, particularly on the diaphragm surfaces, has a tendency for greater blood loss due to greater use of blunt dissection. Operative time was significantly less in the laparoscopic group, which was a surprising finding. This likely reflects selection bias in terms of disease location amenable to laparoscopic resection versus open, despite attempting to control for PCI. Also, while PCI was limited to $\le 10$ for both groups, the median PCI was still lower for the laparoscopic group. The reduced time necessary for incisional closure around HIPEC catheters and at the end of the procedure may also be contributors.

The association of the laparoscopic approach with improved DFS on univariate analysis was most likely the result of selection bias, as there was no association when adjusting for other factors. The only independent predictor of both poorer DFS and OS was CC-score > 0, highlighting the importance of complete cytoreduction, regardless of surgical approach. These findings were essentially the same when the analyses were completed as intention-to-treat, suggesting no downside to starting these procedures laparoscopically. In many of these converted cases, the open incision was directed over the disease identified laparoscopically, and was smaller than the standard xiphoid-to-pubis open incision. This may be why overall inpatient morbidity remained statistically significantly lower in the laparoscopic group in this analysis.

Complex cancer operations that are performed laparoscopically have expanded in recent decades, to include procedures like gastrectomy with D2-node dissection, proctectomy, and pancreatectomy [11–14]. These procedures can be combined with peritonectomy and laparoscopic HIPEC for the treatment of PSM with minimal morbidity. This is the first study to report long-term oncologic outcomes following laparoscopic CRS/HIPEC for patients with PSM inclusive of patients with malignant histologies. Hesitation in performing laparoscopic CRS/HIPEC likely stems from concern for missing peritoneal disease that might otherwise be identified with open surgery. In this study, no patients with LAMN experienced recurrence or disease-related death, and those with high-grade disease did not experience recurrence or death from disease at a rate different than what would be expected with open surgery. CRS/HIPEC for aggressive primary cancers is unlikely to be curative, but rather can improve symptoms and prolong survival, so every effort should be made to minimize the morbidity of the procedure.

This study is limited by a relatively small and mixed cohort of patients, but it demonstrates that clinical trials of laparoscopic CRS/HIPEC are feasible and can direct practice change with even a small number of patients. It also showed that laparoscopic CRS/HIPEC is safe and results in decreased postoperative morbidity compared to open surgery, with no apparent compromise in long-term oncologic outcomes. A logical next step would be to conduct a non-inferiority trial for patients with PSM of specific primary histologies, utilizing this standardized technique for laparoscopic exploration and surgery.
